# Drought Tolerance and Competition in Eastern Redcedar (*Juniperus virginiana*) Encroachment of the Oak-Dominated Cross Timbers

**DOI:** 10.3389/fpls.2020.00059

**Published:** 2020-02-07

**Authors:** Patricia R. Torquato, Chris B. Zou, Arjun Adhikari, Henry D. Adams, Rodney E. Will

**Affiliations:** ^1^Department of Natural Resource Ecology and Management, Oklahoma State University, Stillwater, OK, United States; ^2^School of Ecosystem and Forest Sciences, The University of Melbourne, Burnley, VIC, Australia; ^3^Department of Plant Biology, Ecology, and Evolution, Oklahoma State University, Stillwater, OK, United States

**Keywords:** water potential, stomatal conductance, *Quercus stellata*, mixed forest, encroachment, Great Plains

## Abstract

On the dry, western edge of the eastern deciduous forest of the USA (Cross Timbers), the drought-tolerant, evergreen eastern redcedar (*Juniperus virginiana)* is encroaching into post oak- *(Quercus stellata)* dominated woodlands. The overall goal of this study was to examine whether the drought tolerance strategies of eastern redcedar provide it a competitive advantage over post oak and whether this is a key attribute facilitating its successful establishment in the Cross Timbers. Specifically, we assessed xylem water potential and leaf gas exchange of these two species growing in single-species stands and in a mixed-species stand. We found that both species exhibit a similar degree of isohydry and close their stomates to the same extent in response to declining xylem water potentials. Both species had similar relative reductions in gas exchange in response to drought, despite differences in xylem anatomy. However, post oak had leaf-level gas exchange rates approximately 5× greater than eastern redcedar during periods of high moisture availability. Therefore, it did not appear that eastern redcedar encroachment into an oak-dominated forest is facilitated by growing season differences in carbon gain, although evergreen eastern redcedar can conduct gas exchange year-round when conditions are favorable while post oak is deciduous. We found that volumetric soil water content (0–45 cm) was lower in the pure eastern redcedar stand than the mixed-species or pure post oak stand which may indicate that eastern redcedar may experience favorable soil moisture conditions when encroaching into open oak woodlands. Moreover, water potentials in eastern redcedar tended to be more negative in pure stands compared to the mixed stand. Our results suggest the two species may be using water from different depths, reducing competition. Overall, our findings indicate that eastern redcedar encroachment into formerly oak-dominated Cross Timbers forests likely will continue under moderate drought, in the absence of fire, with consequences for water budgets, carbon cycling, grazing forage, wildlife habitat, and wildfire risk.

## Introduction

Fire exclusion has led to encroachment of trees and shrubs into grassland, savanna, woodland, and forest ecosystems around the world (e.g., Scholes and Archer, 1997; Staver et al., 2011; Schriver et al., 2018). Throughout the eastern USA, fire-intolerant, mesic tree species have proliferated into oak (*Quercus* spp.) woodlands and forests (Nowacki and Abrams, 2008). This alters fuel conditions and understory microenvironment to reduce the likelihood of future fire, i.e., mesophication (Nowacki and Abrams, 2008; Kreye et al., 2018). In addition to altering future fire behavior and species composition, this forest densification process has many ecological impacts such as reducing understory productivity (Feltrin et al., 2016), plant biodiversity (van Els et al., 2010), water yield (e.g., McLaughlin et al., 2013), and wildlife habitat for savanna and woodland species.

Covering almost five million hectares across parts of Kansas, Oklahoma, and Texas (Therrell and Stahle, 1998), the Cross Timbers region is a complex mosaic of woodlands and forest vegetation located on the dry western edge of the eastern deciduous forest (Therrell and Stahle, 1998; Anderson et al., 2007). Cross Timbers forests are dominated by post oak (*Q. stellata*). However, the encroachment of eastern redcedar (*Juniperus virginiana*) is transforming oak-dominated savannas and woodlands into mixed oak/juniper closed-canopy forests (DeSantis et al., 2011; Hoff et al., 2018a). In contrast to the encroachment of mesic hardwoods into oak forests further east, the addition of highly flammable, evergreen eastern redcedar into the midstory of Cross Timbers increases fuel loading and risk of wildfire (Hoff et al., 2018b). During the past century, the changes in woody species composition within the Cross Timbers have been largely associated with fire exclusion and episodic drought (Rice and Penfound, 1959; Burton et al., 2010; DeSantis et al., 2010). As opposed to the increase of mesic hardwoods further east, the encroachment of the drought-hardy eastern redcedar into the Cross Timbers is likely associated with relatively low annual precipitation (average of 660 mm) across Cross Timbers region (Dyksterhuis, 1948; Anderson et al., 2007).

Compared to most other tree species found in the central Great Plains, eastern redcedar is better adapted to water-limited environments (Bahari et al., 1985; Maherali et al., 2006; Volder et al., 2013). Eastern redcedar has extremely narrow tracheids which can withstand high xylem tensions without cavitation and this contributes to eastern redcedar’s ability to tolerate low soil moisture conditions (Eggemeyer et al., 2006; Kannenberg et al., 2019). The species can maintain 50% of its hydraulic conductivity down to relatively low xylem water potentials of −4.5 MPa in the roots and −7.1 MPa in the shoots (Maherali et al., 2006). In addition, its relatively high leaf-level water use efficiency under water stress conditions (Eggemeyer et al., 2006) likely makes eastern redcedar trees highly adapted to water-limited conditions (Eggemeyer et al., 2006; Bihmidine et al., 2010) once fire is eliminated from the landscape. Though post oak is considered drought tolerant when compared to other oaks (Stranskey, 1990), the species has been reported to have relatively low leaf-level water use efficiency under water stress in a greenhouse experiment (Ni and Pallardy, 1991).

Water use strategies of woody plants are crucial for their competitive ability and survival during drought. Plants can be characterized along the an/isohydry spectrum based on strategies to conserve water loss and maintain carbon assimilation (Tardieu and Simonneau, 1998; Klein, 2014), and these strategies interact with the environment conditions (Hochberg et al., 2018). Relatively isohydric species respond to water stress by closing stomata to reduce daily and seasonal fluctuations in water potential, reducing photosynthesis. While carbon gain is reduced, this may reduce the risk of xylem embolism. In contrast, the stomata of relatively anisohydric species are less sensitive to water stress and these species may continue photosynthesis during dry periods though possibly increasing the risk of xylem embolism (Tardieu and Simonneau, 1998). However, the link between degree of an/isohydry and embolism avoidance is not yet clear, as it can be strongly mediated by differences in hydraulic vulnerability among species (Garcia-Forner et al., 2016). Therefore, a species such as eastern redcedar which has xylem highly resistant to embolism and an ability to keep stomata open under declining water availability should have a carbon gain advantage in the drought-prone Cross Timbers by exhibiting anisohydric behavior. However, the physiological mechanism remains unknown regarding how the shade-intolerant, evergreen eastern redcedar is able to grow under the oak canopy and widely establish in the midstory in the water-limited Cross Timbers region.

In addition to differing leaf-level drought tolerance strategies among post oak and eastern redcedar, niche differentiation may also play a role in facilitating eastern redcedar encroachment. Differences in rooting depth or structure reduce competition for water among trees and grasses (e.g., Ward et al., 2013). Likewise, when comparing tree species, interspecific differences in rooting depth can be correlated with water relations (Nardini et al., 2016) and a recent review found that in the majority of instances, increasing tree species richness mitigated drought effects (Grossiord, 2019). Given the differences in rooting depth among eastern redcedar and oak (Hinckley et al., 1981), resource partitioning may reduce competition for water in mixed-species stands as compared to pure-species stands and help facilitate eastern redcedar encroachment into the oak-dominated Cross Timbers woodlands.

The overall goal of this study was to examine whether the drought tolerance strategies of eastern redcedar provide it a competitive advantage over post oak and whether this is a key attribute facilitating its successful establishment in the Cross Timbers. Specifically, we assessed the dynamics of volumetric soil water content (θ), xylem water potential (Ψ), net photosynthetic rates (P_n_), stomatal conductance (*g*_s_), and water use efficiency (WUE) for these two species growing in single-species stands and in a mixed-species stand. We hypothesized: (1) compared to post oak, eastern redcedar exhibits a greater degree of anisohydric behavior during low soil moisture conditions such that leaf gas exchange is maintained at the expense of more negative xylem water potentials, and (2) when growing in mixed stands, interspecific competition for water between post oak and eastern redcedar and its effects on leaf gas exchange are smaller than intraspecific competition for water when growing in single-species stands.

## Materials and Methods

### Study Site

The study was conducted at the Cross Timbers Experimental Range, a research and outreach facility owned by Oklahoma State University, 15 km southwest of Stillwater, Payne County, Oklahoma, USA (latitude 36°04'05''N, longitude 97°11'25''W). The site selected for the study was dominated by mature post oaks ranging from 6.2 to 30.7 cm diameter at breast height (DBH) and mature eastern redcedars ranging from 7.2 to 31.4 cm DBH (**Table 1**). The elevation is 331 m above the sea level, and the mean annual temperature is 15.5°C. The soil is the Stephenville series (Fine-loamy, siliceous, active, thermic Ultic Haplustalfs) consisting of moderately deep (approximate 1 m), well drained soils weathered from sandstone. The soil is mostly loamy fine sand in the upper 40 cm and sandy clay loam below 40 cm (Soil Survey Staff, 2018). Based on the Oklahoma Mesonet Marena Station located 2.1 km from the study site, the annual average precipitation is 887.9 mm with 66% falling during the growing season (April–September; Oklahoma Climatological Survey, 2018).

**Table 1:** Mean diameter at breast height (DBH), basal area (BA), and tree density for each stand for stems greater than 5 cm DBH.

Stand	Species	DBH (cm ± SD)	BA (m^2^ ha^-1^)	Tree density (n/ha-1)
OAK	Post oak	18.6 (± 4.5)	17.5	609
	Redcedar	11.4 (± 3.0)	1.6	152
	Other	11.8 (± 1.9)	1.3	114
	Total		20.5	876
MIX	Post oak	19.9 (± 10.1)	8.9	229
	Redcedar	14.8 (± 8.2)	9.3	419
	Blackjack oak	13.1 (± 7.8)	1.5	190
	Other	6.9 (± 0.9)	0.4	114
	Total		20.1	952
ERC	Redcedar	13.7 (± 7.4)	16.0	838
	Post oak	18.9 (± 0.0)	1.1	38
	Blackjack oak	14.4 (± 6.0)	2.2	114
	Other	10.0 (± 0.0)	0.3	38
	Total		19.5	1028

Each stand had an area of 262.5 m^2^ (0.026 ha).

### Experimental Design

To test our hypotheses, we conducted a 17-month, field-based experiment spanning two growing seasons. In the early spring of 2017, we selected three stands representing different proportions of oak and eastern redcedar. The first stand was composed mainly of post oaks (OAK stand; 85% of basal area was post oak), the second stand was composed primarily of eastern redcedars (ERC stand; 82% of basal area was eastern redcedar), and the third stand was composed of post oaks (44% of basal area) and eastern redcedars (46% of basal area; MIX stand) (**Table 1**). Each stand was a circle with a 9.1 m radius, totaling 262.5 m^2^. Stands were less than 60 m apart from one another. Stands were selected based on a similarity of basal area and tree density (**Table 1**). Eastern redcedars and post oaks representing the full range of DBH were identified in each stand for physiological measurements. There were seven post oaks selected within the OAK stand, seven eastern redcedars selected within the ERC stand, and five post oaks and five eastern redcedars selected within the MIX stand. The mean (± SD) basal area of the three different stands was 20.02 ± 0.39 m^2^ ha^-1^. Measured at breast height, the mean age (± SD) of the post oaks was 95.7 ± 17.6 y, ranging between 56 and 120 y, and the mean age of eastern redcedar was 45.6 ± 12.5 y, ranging between 36 and 69 y.

### Microclimate and Soil Moisture

In the early spring of 2017, three locations at the OAK stand, three locations at ERC stand, and five locations at the MIX stand were established to monitor soil moisture. For each location, one pair of 15 cm long stainless steel rods (3.2 mm in diameter) were inserted 3 cm apart perpendicular to the soil surface. A second pair of 45 cm long rods were inserted close to the first pair. Volumetric soil water contents (θ) for soil depths between 0–15 cm and 0–45 cm were estimated using a 1502B Metallic Cable Tester (Tektronix, Inc., Beaverton, Oregon, USA), following the methodology of Evett (2003). Measurements were taken every 15 days from April 2017 to September 2018, except from between October 2017 to March 2018 when measurements were taken every 30 days. The θ for the 15–45 cm layer was calculated by subtracting the soil water from the 0–15 cm layer from the 0–45 cm layer weighting the values based on profile depths. Daily precipitation, solar net radiation, air temperature, humidity, and vapor pressure deficit (VPD) were collected from the Marena Station of the Oklahoma Mesonet which was 2.1 km from the study site (https://www.mesonet.org/index.php/weather/daily_data_retrieval). The Palmer Drought Severity Index, a site-specific measure of dryness based on temperature and precipitation that typically ranges from −4 to 4 (Dai and National Center for Atmospheric Research Staff, 2019), was used to estimate relative dryness of the area where the study site is located.

### Physiological Responses

We measured midday xylem water potential (Ψ_M_), leaf net photosynthetic rate (P_n_), and stomatal conductance (*g*_s_) of all selected eastern redcedar and post oak trees bi-weekly from April to September in both 2017 and 2018. From October 2017 to March 2018, monthly measurements of the eastern redcedar trees were conducted. Predawn xylem water potentials (Ψ_P_) were measured monthly for all selected trees during the growing season and for eastern redcedars during winter. To determine xylem water potential, shoots were collected from the mid-canopy of each tree with a pole pruner and stored in individual plastic bags in a cooler until the measurement was performed (less than 30 min). Shoots for Ψ_P_ measurement were collected before sunrise and shoots for Ψ_M_ measurement were collected between 1100 and 1300. Xylem water potentials were measured in the field using a Scholander pressure chamber (Soil Moisture Equipment Corp., Model 3005, Santa Barbara, CA).

Net photosynthesis and *g*_s_ were measured using the LI-COR 6400 portable photosynthesis system (LI-COR, Inc., Lincoln, NE) for leaves on shoots clipped using a pole pruner. Prior to clipping, leaves were in the mid to upper canopy and mostly exposed to full sunlight. Gas exchange was measured immediately after clipping. A 2 × 3 cm opaque chamber (model 6400-02B, LICOR, Inc., Lincoln, NE) with a red/blue LED light source was used with photosynthetically active radiation (PAR) set to 1,800 µmol m^-2^ s^-1^. Carbon dioxide concentration entering the cuvette was set at 400 ppm and conditions of ambient temperature and humidity were maintained inside the cuvette. Once stabilized, four successive measurements were taken at 2-s intervals and averaged for further analyses. Measurements were made between 1000 and 1400 for all 24 trees on the same day for each collection date. Final calculations of P_n_ and *g*_s_ were based on leaf area. After gas exchange measurement of eastern redcedar, the foliage sample in the cuvette was excised and stored in paper envelopes and then dried in an oven at 60°C for 7 days to obtain dry weight. The equation from Cregg (1992) was used to calculate the leaf area from the dry weight. No calculations were necessary for post oaks as their leaves were large enough to cover the entire leaf chamber.

Intrinsic water use efficiency (WUE) was calculated for each gas exchange measurement as the ratio of P_n_ and corresponding *g*_s_. Intrinsic WUE was chosen instead of instantaneous WUE (P_n_/transpiration rate) because small VPD changes during the measurement period can affect leaf-level transpiration rates (Yi et al., 2017), while *g*_s_ is generally not affected. We assessed the isohydric and anisohydric behavior of redcedar and post oak based on the slope of regression between: 1) predawn and midday water potential values and 2) relativized stomatal conductance and midday water potential values. Relativized *g*_s_ was calculated by dividing the measured *g*_s_ by the maximum *g*_s_ measured for the species.

### Data Analysis

Data were analyzed using SAS 9.4 (SAS Inc., Carey, NC, USA). We tested the following comparisons, 1) post oak growing in the OAK stand vs post oak growing in the MIX stand to determine the effects of intraspecific and interspecific competition on oak physiology, 2) eastern redcedar growing in the ERC stand vs eastern redcedar growing in the MIX stand to determine the effects of intraspecific and interspecific competition on eastern redcedar physiology, 3) post oak in the MIX stand vs eastern redcedar in the MIX stand to determine how the two species differ when growing together. Because eastern redcedar was measured year-round while post oak was only measured during the growing seasons, comparisons involving oak (1 and 3) used growing season data only while the comparison among eastern redcedar (2) included all data. From this point forward, the post oaks at the OAK stand are abbreviated as “Oak_O_” and the post oaks at the MIX stand are abbreviated as “Oak_M_”. Eastern redcedar trees at the ERC stand are abbreviated as “ERC_E_” and the eastern redcedar trees at the MIX stand are abbreviated as “ERC_M_”.

To determine whether Oak_O_ and Oak_M_ differed, ERC_E_ and ERC_M_ differed, and Oak_M_ and ERC_M_ differed, we tested for differences in Ψ_P_, Ψ_M_, P_n_, *g*_s_, and WUE using a PROC MIXED model with repeated measurements and autoregressive covariance structure. Date, species, and stand were considered fixed effects and individual trees were considered a random effect. The repeated θ measurements for each stand were analyzed using PROC MIXED with a similar model, testing for differences in month and stand as well as their interaction. The PDIFF option was used in all analyses to compare the LS-means of different treatment groups. To test whether the relationships between Ψ_P_ and Ψ_M_ differed between redcedar and post oak, analysis of covariance was used to test for differences in slopes or intercepts. Likewise, the relationship between Ψ_M_ and *g*_s_ was tested using *g*_s_ relativized of to the maximum stomatal conductance reading. As we were unable to replicate treatments at the stand level, results comparing ERC_E_ and ERC_M_ and results comparing Oak_O_ and Oak_M_ are specific to the stands used in our study. Comparisons among ERC_M_ and Oak_M_ are more robust as each tree is a replicate.

## Results

### Environmental Conditions

**Figure 1:**
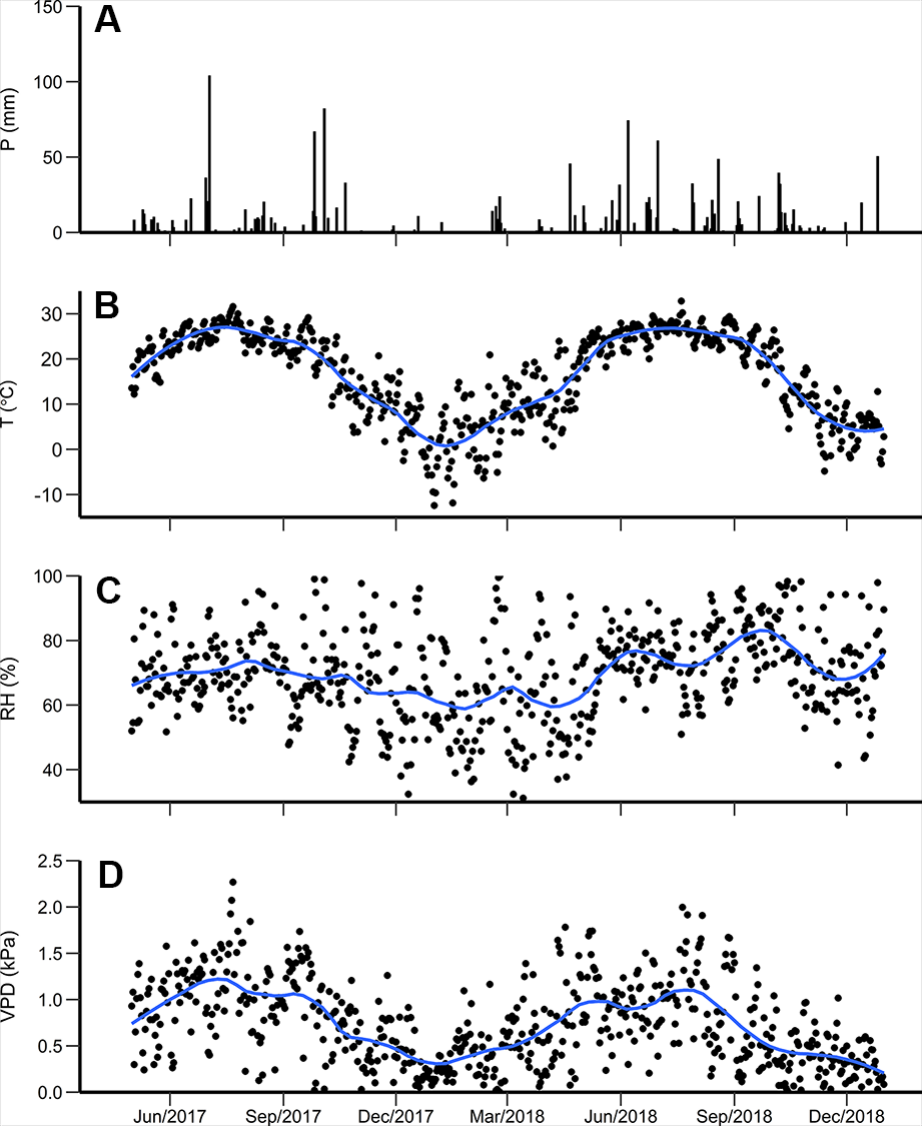
**(A)** Daily precipitation (P), **(B)** average temperature (T), **(C)** relative humidity (RH), and **(D)** vapor pressure deficit (VPD) from April 2017 to September 2018. The blue line is the locally weighted regression using local neighborhood with size of 5 days.

During the study period (April 2017–September 2018), total precipitation was 1,573 mm, with 977 mm in the 12 month period from April 2017 to March 2018 (**Figure 1A**). Throughout the 550 day study period, there were 137 days with rain, of which 69 days had precipitation less than 5 mm and 13 days had precipitation greater than 25 mm. Of the total precipitation measured, 45% occurred during spring (6 months of the total 18 month period). The largest daily precipitation was 125 mm on April 29, 2017. The longest period with no precipitation was 36 days between January 11 and February 15, 2018. From July 4 to September 25, 2017 (84 days), there was only 119 mm of rainfall distributed in events all smaller than 20 mm which contributed to a drought that reached the abnormal classification during September based on the Palmer Drought Severity Index (Dai and National Center for Atmospheric Research Staff, 2017). Additionally, a drought classified as moderate occurred in July 2018 (Dai and National Center for Atmospheric Research Staff, 2019).

During the study period, the daily average temperature (± SD) was 17.8 ± 9.2°C, ranging from −12.4 to 32.8°C. The daily mean temperature for the growing season (April to October) (± SD) was 22.5 ± 9.6°C in 2017 and 22.7 ± 6.0°C in 2018 (**Figure 1B**). The average daily relative humidity was lowest during winter (December 2017 to March 2018), reaching 26.2% on January 25, 2018 (**Figure 1C**). The daily average VPD ranged from 0.003 to 2.7 kPa (**Figure 1D**).

### Volumetric Soil Water Content

**Figure 2:**
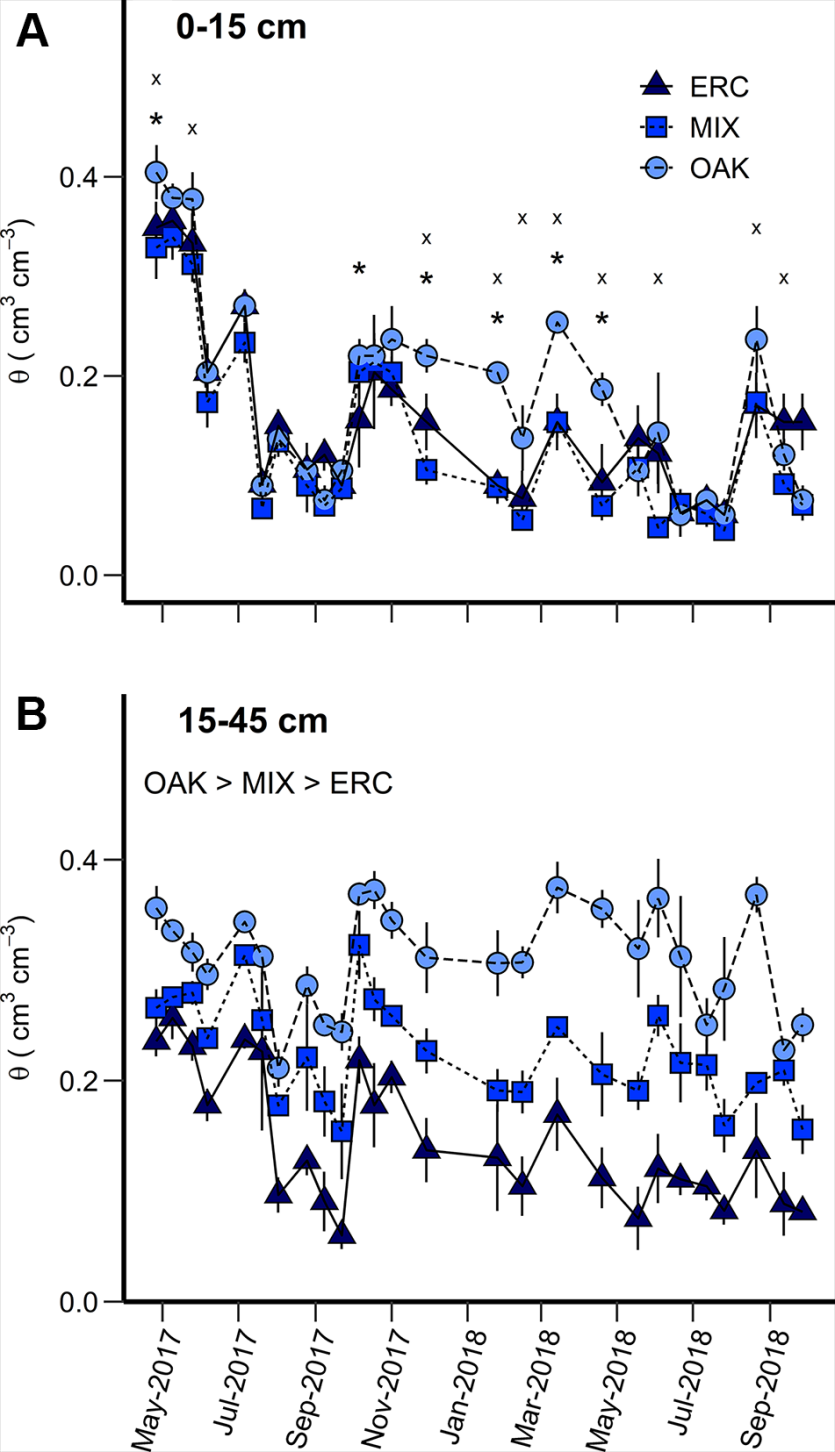
Volumetric soil water content (*θ*) for the **(A)** 0–15 cm and **(B)** 15–45 cm soil layers for the oak stand (OAK), mixed stand (MIX), and eastern redcedar stand (ERC) between April 2017 and September 2018. For the 0–15 cm layer, asterisks (*) indicates significant differences (p < 0.05) between OAK and ERC, and the letter “× “ indicates significant differences (p < 0.05) between OAK and MIX. For the 15–45 cm layer, mean daily *θ* was significantly different among stands (OAK stand > MIX stand > ERC stand) (p < 0.05). Vertical bars represent standard error of measurements at each date (not shown when smaller than the symbol size).

For the 0–15 cm soil layer, θ calculated at the monthly scale varied among months (*p* < 0.0001) and stands (*p* < 0.003) with interactions between month and stand type significant for OAK vs ERC and OAK vs MIX (*p* < 0.05, **Table 2**). These interactions mainly occurred due to greater θ of the OAK stand than the MIX or ERC stands for the months when oaks leaves were absent (*p* < 0.05), i.e., θ of the OAK stand was approximately double that of the ERC and MIX stands in winter (**Figure 2A**). In contrast, θ of the OAK, MIX, and ERC stands were lower and typically similar during the growing season (**Figure 2A**). Across all dates, the average θ of the ERC stand was 14.5% greater than for the MIX stand.

**Table 2:** Results of PROC MIXED model with repeated measurements and autoregressive covariance structure for effects of date, stand, and their interaction on soil water content (*θ*) in 0–15 and 15–45 cm depth. Bold text indicates significance p < 0.05.

Stand	Variable	0–15 cm	15–45 cm
ERC vs MIX	Date	**<0.0001**	**<0.0001**
	Stand	**0.0028**	**<0.0001**
	Date × Stand	0.3604	0.4285
ERC vs OAK	Date	**<0.0001**	**<0.0001**
	Stand	**0.0007**	**<0.0001**
	Date × Stand	**0.0043**	0.072
OAK vs MIX	Date	**<0.0001**	**<0.0001**
	Stand	**<0.0001**	**<0.0001**
	Date × Stand	**0.0024**	0.382

For the 15–45 cm soil layer, θ varied across months (*p* < 0.0001) and stands (*p* < 0.0001) but the interactions between month and stand were not significant (*p* > 0.05, **Table 2**). On average, θ (± SD) of the OAK stand (0.31 ± 0.06 cm^3^ cm^-3^) was greater than θ at the MIX stand (0.23 ± 0.07 cm^3^ cm^-3^) and the ERC stand (0.15 ± 0.07 cm^3^ cm^-3^), while θ at the MIX stand was greater than θ of the ERC stand (**Figure 2B**, **Table 2**). For both years, the lowest θ occurred in the late growing season for all stands. The lowest values of θ for OAK, MIX, and ERC stands were 0.21, 0.12, and 0.06 cm^3^ cm^-3^ in 2017 and 0.25, 0.15, 0.08 cm^3^ cm^-3^ in 2018, respectively.

### Physiological Responses

**Figure 3:**
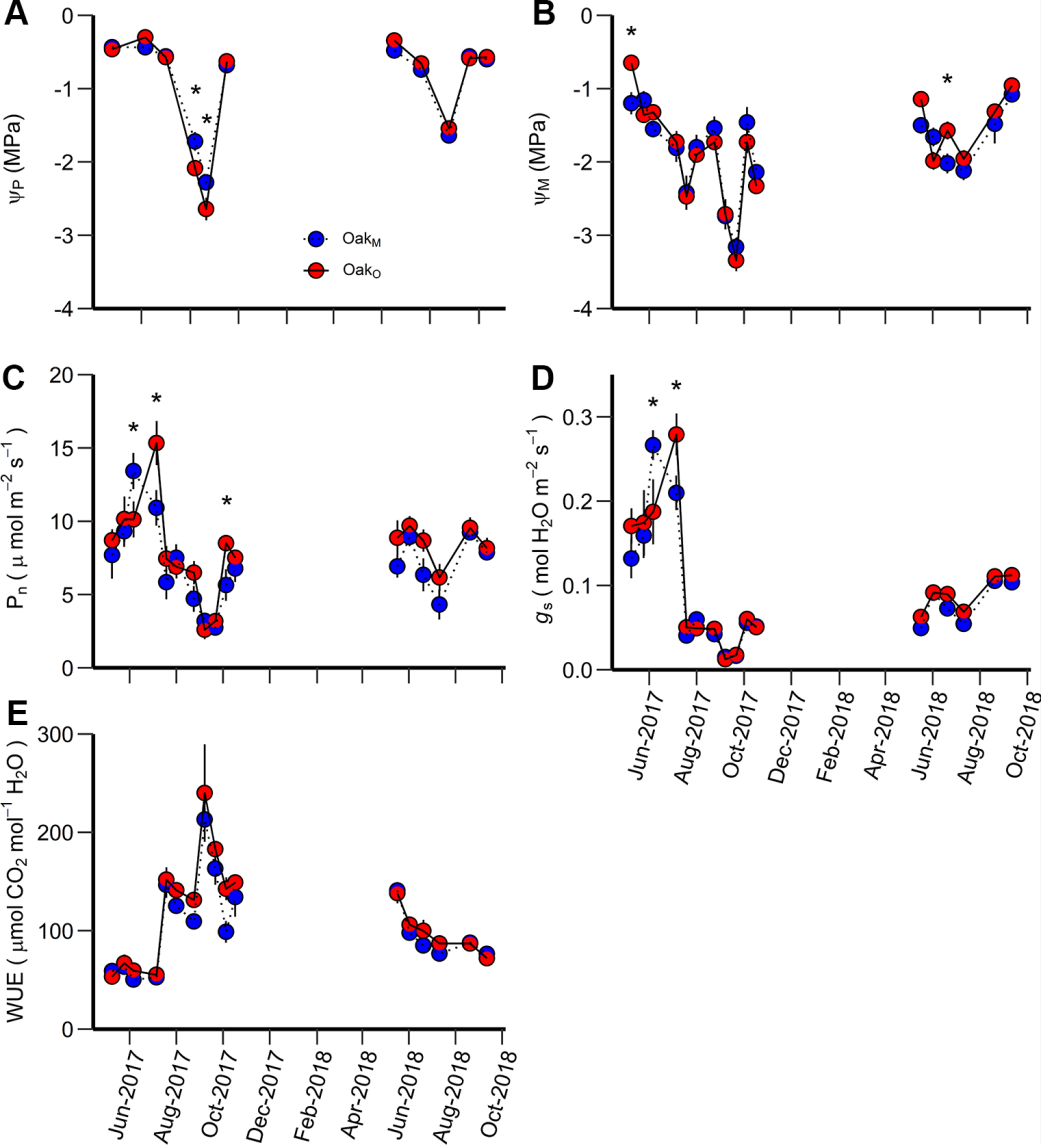
Predawn water potential (Ψ_P_) **(A)**, midday water potential (Ψ_M_) **(B)**, net photosynthetic rate (P_n_) **(C)**, stomatal conductance (g_s_) **(D)**, and water use efficiency (WUE) **(E)** measured during the study period for Oak_M_ vs. Oak_O_. Oak_M_ are post oak trees growing in a mixed-species stand while Oak_O_ are post oak growing in single-species stands. An asterisk (*) indicates significant differences on specific dates (p < 0.05). Vertical bars represent standard errors of measurements at each date (not shown when smaller than the symbol size).

When comparing leaf gas exchange variables among the post oaks growing in mixed- and single-species stands (Oak_O_ vs Oak_M_), Ψ_P_ of Oak_M_ was greater than Oak_O_ during the driest period, but there was no consistency in differences for Ψ_M_, P_n_, and *g*_s_. The differences for Ψ_P_, Ψ_M_, P_n_, and *g*_s_ among post oaks (Oak_O_ vs Oak_M_) depended on the measurement date, i.e., there were significant interactions between date and stand (**Table 3**). For Ψ_P,_ Oak_O_ and Oak_M_ differed when Ψ_P_ was more negative than −1.6 MPa which occurred during the late growing season of 2017 when the overall Ψ_P_ and the soil moisture were very low (**Figures 2**, **3A**). Comparing Ψ_M_ of post oak in different stands, Oak_O_ had significantly higher (less negative) Ψ_M_ than Oak_M_ during the early growing season of 2017 when soil moisture was high and in July 2018 (**Figures 2**, **3B**). The P_n_ of Oak_O_ was greater than Oak_M_ on two dates (August 2017 and October 2017) and for *g*_s_ on one date (August 2017), while Oak_M_ was greater for both P_n_ and *g*_s_ on one date (June 2017) (**Figures 3C, D**). Net photosynthesis and stomatal conductance followed the same trends and exhibited a decrease of 76% in P_n_ and 93% in *g*_s_ from June to September 2017 corresponding to the abnormal drought period. The WUE of Oak_O_ and Oak_M_ did not significantly differ among the stands and exhibited the highest values during the drought period of 2017 (**Table 3**, **Figure 3E**).

**Table 3:** Results of PROC MIXED model with repeated measurements for the effects of date, stand, or species, and their interaction on gas exchange and water potential. Bold text indicates significance p < 0.05.

Species	Variable	P_n_	*g*_s_	Ψ_P_	Ψ_M_	WUE
Oak_O_ vs Oak_M_	Date	**<0.0001**	**<0.0001**	**<0.0001**	**<0.0001**	**<0.0001**
	Stand	0.0724	0.0877	0.5425	0.878	0.0805
	Date × stand	**0.0463**	**0.0154**	**0.008**	**0.006**	0.9400
ERC_E_ vs ERC_M_	Date	**<0.0001**	**<0.0001**	**<0.0001**	**<0.0001**	**<0.0001**
	Stand	0.2153	0.4241	0.8168	0.1078	0.4857
	Date × stand	0.2997	0.3076	**0.0002**	**<0.0001**	0.1302
Oak_M_ vs ERC_M_	Date	**<0.0001**	**<0.0001**	**<0.0001**	**<0.0001**	**<0.0001**
	Species	**<0.0001**	**<0.0001**	**<0.0001**	**0.0302**	0.1889
	Date × species	**<0.0001**	**<0.0001**	**<0.0001**	**<0.0001**	**0.0010**

P_n_ is net photosynthesis, g_s_ is stomatal conductance, Ψ_P_ is predawn water potential, Ψ_M_ is midday water potential, and WUE is water use efficiency. Oak_O_ are post oak growing in a stand dominated by post oak, Oak_M_ are post oak growing in mixed-species stands, ERC_E_ are eastern redcedar growing in a stand dominated by eastern redcedar, and ERC_M_ are eastern redcedar growing in a mixed stand.

**Figure 4:**
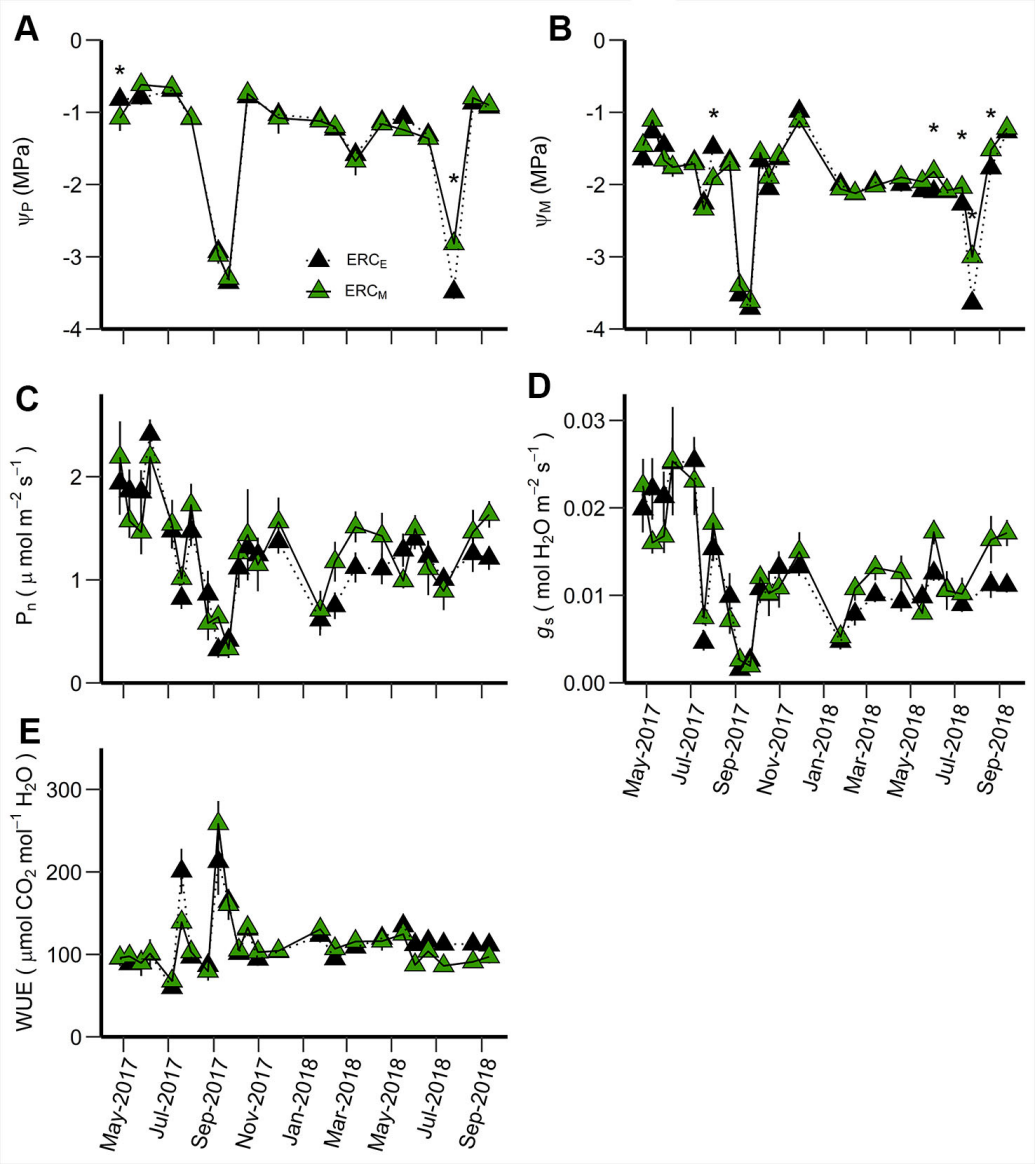
Predawn water potential (Ψ_P_) **(A)**, midday water potential (Ψ_M_) **(B)**, net photosynthetic rate (P_n_) **(C)**, stomatal conductance (g_s_) **(D)**, and water use efficiency (WUE) **(E)** for ERC_M_ vs. ERC_E_. ERC_M_ are eastern redcedar trees growing in a mixed-species stand while ERC_E_ are eastern redcedar trees growing in single-species stands. An asterisk (*) indicates significant differences on specific dates (p < 0.05). Vertical bars represent standard errors of measurements at each date (not shown when smaller than the symbol size).

Among leaf gas exchange variables comparing eastern redcedar growing in mixed- and single-species stands, only water potentials were significantly different and only during the summer (date × stand interaction) (**Table 3**). The differences in Ψ_P_ between ERC_E_ and ERC_M_ were generally small with lower Ψ_P_ for ERC_M_ in May 2017 and lower Ψ_P_ for ERC_E_ in July 2018 (**Figure 4A**). For Ψ_M_, there was one date in 2017 where ERC_M_ was lower than ERC_E_, but in 2018, there were four dates during the middle of the growing season where ERC_E_ was lower than ERC_M_ (**Figure 4B**). The highest values for P_n_ occurred during June 2017 and the highest value for *g*_s_ in July 2017 (**Figures 4C, D**). The lowest values for both P_n_ and *g*_s_ occurred during September 2017, when WUE was the highest (**Figure 4E**).

**Figure 5:**
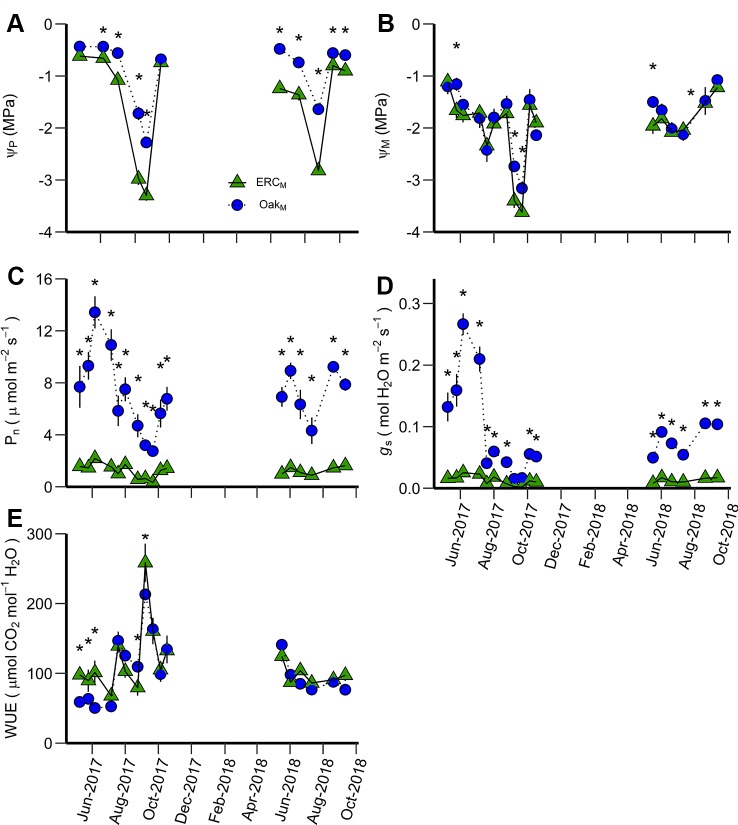
Predawn water potential (Ψ_P_) **(A)**, midday water potential (Ψ_M_) **(B)**, net photosynthetic rate (P_n_) **(C)**, stomatal conductance (g_s_) **(D)**, and water use efficiency (WUE) **(E)** for ERC_M_ vs. Oak_M_. ERC_M_ and Oak_M_ are eastern redcedar and post oak trees growing in a mixed-species stand. An asterisk (*) indicates significant differences on specific dates (p < 0.05). Vertical bars represent standard errors of measurements at each date (not shown when smaller than the symbol size).

The leaf-level variables Ψ_P_, Ψ_M_, P_n_, *g*_s_ were greater for post oak than eastern redcedar in the mixed stand (Oak_M_ vs ERC_M_) (significant species effect, **Table 3**). Although there were significant interactions between date and species, these generally occurred because the magnitude of differences varied over time. The Ψ_P_ of ERC_M_ was significantly more negative (*p* < 0.05) than that of Oak_M_ on nine of eleven dates, all except for the first and last measurement of 2018 (**Figure 5A**). The Ψ_M_ of the ERC_M_ was significantly more negative than Oak_M_ during the drier sampling periods (5 out of 17 measurement date) (**Figure 5B**). Both P_n_ and *g*_s_ of the Oak_M_ were significantly greater than the ERC_M_ for the all data collections, except for a late growing season date in 2017 when the entire soil profile was very dry (**Figures 5C, D**). In addition, WUE of the Oak_M_ was significantly lower than ERC_M_ during May and June 2017 and again in October 2017 (**Figure 5E**).

**Figure 6:**
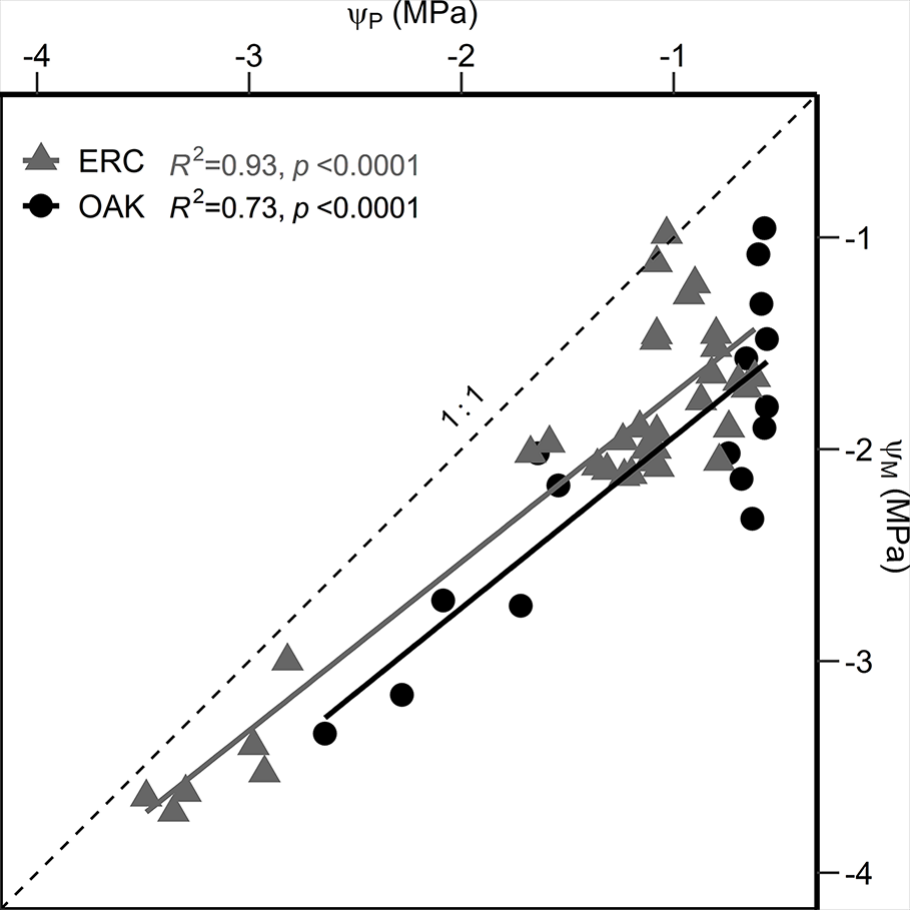
Linear regressions between the predawn water potential (Ψ_P_) and midday water potential (Ψ_M_) for post oak (circles) and eastern redcedar (triangles). The 1:1 relationship is indicated by a dashed line. Equations are as follow: Ψ_M_ oak = 0.80*Ψ_P_ − 1.13; Ψ_M_ erc = 0.79*Ψ_P_ − 0.99.

**Figure 7:**
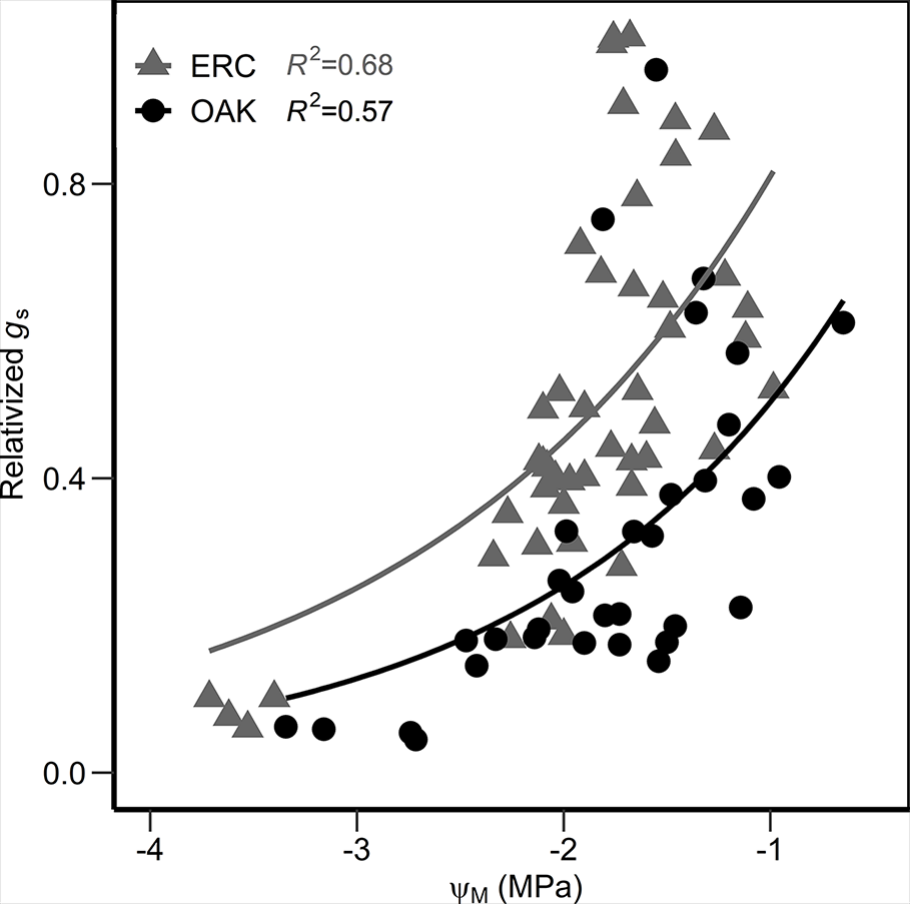
Relationship between the relativized stomatal conductance (measured g_s_ divided by the maximum stomatal conductance reading of each species, relativized g_s_) and the midday water potential (Ψ_M_) for post oak (circles) and eastern redcedar (triangles). Equations are as follow: g_s_ oak = 1.47 e^0.97^ ^Ψ^; g_s_ erc = 2.33 e^0.89Ψ^.

When the relationship between Ψ_M_ and Ψ_P_ were compared, the slopes for post oak (0.80 ± 0.11 SE) and eastern redcedar (0.79 ± 0.05 SE) were similar (**Figure 6**), with no significant difference in slope or intercept (*p* > 0.05). Comparing the two species at Ψ_P_ of −1.0 MPa, the corresponding Ψ_M_ were −1.93 and −1.72 MPa for post oak and eastern redcedar. Comparing at Ψ_P_ of −3 MPa, the corresponding Ψ_M_ were −3.53 and −3.30 MPa, respectively. The relationship between relativized *g*_s_ and Ψ_M_ (**Figure 7**) exhibited a similar nonlinear relationship for both post oak and eastern redcedar, indicating the decrease in *g*_s_ with increasingly negative Ψ_M_ was consistent for both species. Therefore, even though eastern redcedar tended to have lower water potentials than post oak, the degree to which stomata close per unit change in Ψ_M_ was similar.

## Discussion

In this study, we evaluated if eastern redcedar possesses physiological characteristics that enable its encroachment in a post oak dominated forest within the Cross Timbers region. Our study found that both post oak and eastern redcedar respond similarly to water potential decrease by closing stomata. Post oak exhibited higher gas exchange rates (both photosynthesis and stomatal conductance) compared to the eastern redcedar when there was adequate soil moisture during the growing season. While leaf-level rates of P_n_ and *g*_s_ were approximately 5× greater in post oak than eastern redcedar, both species exhibited relative decreases of similar magnitude during drought periods. Soil water contents were greater in a pure oak stand than in stands including eastern redcedar which could facilitate eastern redcedar encroachment into areas currently dominated by post oak. We found that water potentials were occasionally more negative for post oak and eastern redcedar growing in single-species vs mixed-species stands, likely indicating greater intraspecific competition for water in pure stands.

The slope of relationship between Ψ_P_ and Ψ_M_ can be used to evaluate the water stress response strategy of plants along the anisohydric/isohydric spectrum (Martínez-Vilalta et al., 2014). Following the classification scheme of Martínez-Vilalta et al. (2014), a slope equal to 0 for the Ψ_M_/Ψ_P_ relationship indicates strict isohydry, a slope between 0 and 1 indicates partial isohydry, a slope equal to 1 indicates strict anisohydry, and a slope greater than 1 indicates extreme anisohydry. Both species had nearly identical slopes less than 1, i.e., 0.79 and 0.80, indicating both are partially isohydric. The slopes between Ψ_M_ and Ψ_P_ for our two species were close to the average slope (0.86) for 104 species compiled by Martínez-Vilalta et al. (2014). Given the similarity in slopes and intercepts for eastern redcedar and post oak, there was no evidence in our study to support the hypothesis that eastern redcedar is more anisohydric than post oak.

In addition to evidence from the Ψ_M_/Ψ_P_ relationship, similar relationships between relativized *g*_s_ and Ψ_M_ of post oak and eastern redcedar in our study indicate a similar response of stomata of both species to water stress. Likewise, the absolute value of *g*_s_ in both species approached zero during the driest period, further supporting that both species readily close their stomata during drought. A study during a severe drought in Missouri, USA found that both eastern redcedar and *Q. alba*, a species closely related to post oak, exhibited stomatal closure early in the drought period compared to other co-occurring species which led the authors to speculate that eastern redcedar and *Q. alba* prioritize stomatal closure as a drought-avoidance mechanism (Hinckley et al., 1979).

These similarities in response to soil drying among post oak and eastern redcedar occur even though the species have very different xylem anatomy. *Juniperus* spp. have xylem composed of extremely narrow tracheids ranging from 8 to 9.5 µm which enable them to withstand low water potentials without cavitating (McElrone et al., 2004; Maherali et al., 2006; Pittermann and Sperry, 2006). Post oaks have xylem composed of vessels and tracheids ranging from 14.9 to 27.2 µm diameter which should make them more vulnerable to embolism (Cavender-Bares and Holbrook, 2001). The similarity in *g*_s_ response to soil drying for the two species with very different xylem anatomy seems to indicate the importance of stomatal control as the dominant mechanism to withstand the moderate drought in the relatively xeric Cross Timbers forest we measured.

Our experiment was performed during moderate drought, which did not irreversibly damage the leaf physiological activity of either species. The xylem morphology of eastern redcedar enables it to withstand xylem water potential to at least −7.1 MPa (Maherali et al., 2006), which is much lower than the most negative xylem water potential recorded during our study. In contrast, post oak in greenhouse studies can show early signs of turgor loss at leaf water potentials between −2.0 and −2.8 MPa (Parker and Pallardy, 1988; Kwon and Pallardy, 1989). However, the lower Ψ_M_ of post oak than these values in our study likely indicates that mature, field-grown post oak trees can withstand lower water potential or that post oak can alter its turgor loss point through osmotic adjustment during drought (Parker and Pallardy, 1988). Notably, post oak in our study experienced midday water potentials as low as −3.2 to −3.4 MPa in September 2017 (for MIX and OAK stands, respectively), yet photosynthesis increased following rainfall in October 2017. Under extreme drought, post oak undergoes early leaf senescence (Baldocchi and Xu, 2007) which likely helps to prevent embolism while eastern redcedar likely relies on its extreme resistance to cavitation associated with its very narrow tracheids.

In our study, P_n_ and *g*_s_ of post oak were approximately fivefold greater than eastern redcedar during the growing season which is consistent to findings of Volder et al. (2010) where post oak seedlings exhibited two times greater P_n_ and *g*_s_ than eastern redcedar. Similarly, Owens (1996) reported twofold greater *g*_s_ and 30% greater P_n_ of *Q. virginiana* than that of *J. ashei* (closely related to *J. virginiana*). Part of what drives these differences is the basis for gas exchange calculation. The specific leaf area (cm^2^ g^-1^) of eastern redcedar was approximately 116 cm^2^ g^-1^ when calculated on an all-sided basis (Cregg, 1992). In contrast, post oak specific leaf area in our study was measured 55 cm^2^ g^-1^. If calculated on a weight rather than an area basis, this would roughly halve the difference in gas exchange among species.

Our second hypothesis predicted lower competition for water in the mixed-species stand than in the pure species stands which would be supported by higher θ and Ψ when comparing individuals in the MIX stand to the individuals in the OAK and ERC stands. Consistent with this hypothesis, we found that Ψ_P_ for post oak was higher in the MIX stand than the OAK stand under moderate water stress likely indicating more intense competition for water in pure oak stands. Furthermore, Ψ_M_ tended to be lower for eastern redcedar in the ERC stand than in the MIX stand. However, these differences were not consistent across the two growing seasons. Volumetric soil content was greater in the MIX stand than the ERC stand (53% greater for the 15–45 cm layer) indicating that eastern redcedar trees growing with post oak probably have greater soil moisture available than when growing by themselves. Assessment of the effects of θ on competition among post oak growing in the OAK and MIX stands is difficult because while there is clearly lower θ in the MIX than OAK stand, likely due to year-round water use of eastern redcedar in the MIX stand, post oak accesses water below the depth we measured (45 cm). Assuming the encroaching eastern redcedar are primarily rooted in the upper soil layers (Hinckley et al., 1981), then there may be greater θ in the MIX than the OAK stand below 45 cm.

The effects of interspecific competition for soil water can be less intense than intraspecific competition if there are differences in rooting structure and rooting depth among species (Davis et al., 1998; Nardini et al., 2016). Eastern redcedar is relatively shallow-rooted (upper 1 m) compared to co-occurring oak species (Hinckley et al., 1981). In contrast, *Quercus* spp. are among the most deeply rooted trees in North America (Abrams, 1990) and more evenly distribute their roots throughout the upper several meters of soil (Hinckley et al., 1981). Also, a plastic response of rooting depth among competing species may occur to perhaps avoid direct interspecific competition for water. For instance, the rooting structure of *Fagus sylvatica* was shallower when growing in combination with *Q. petraea* than when growing in pure stands (Leuschner et al., 2001). In addition to reduced competition for water due to resource partitioning in mixed species stands, oak might facilitate encroachment of eastern redcedar through redistribution of water from deeper depths. *Quercus* spp. can exhibit hydraulic lift which may facilitate water acquisition by other more shallowly rooted species (Zapater et al., 2011). If hydraulic redistribution is occurring, it is likely asymmetric as *Juniperus* spp. have shown less ability to redistribute water than other species (Armas et al., 2010), and because eastern redcedar is shallowly rooted.

We found that both post oak and eastern redcedar respond similarly to soil drying by closing stomata, but that post oak has much greater rates of photosynthesis during times of adequate soil moisture. Therefore, we did not find physiological evidence that eastern redcedar, in the absence of fire, is encroaching into the Cross Timbers forest we measured through more favorable carbon gain during drought. Our study showed that eastern redcedar may benefit from greater soil moisture availability when encroaching into oak stands than when growing in pure stands which may facilitate encroachment into oak woodlands. However, we chose stands of similar basal area such that we measured a substitution of eastern redcedar for oak when comparing the MIX to the OAK stand. A denser oak stand may use more water throughout the soil profile and negate the increased soil moisture response we measured in the MIX stand. Rather than drought tolerance, the evergreen nature of eastern redcedar may facilitate its encroachment. Lassoie et al. (1983) found that eastern redcedar saplings growing under a closed-canopy of oak had the highest rates of carbon gain in spring before overstory canopy development. In a location adjacent to the study site, eastern redcedar transpired water year-round (Caterina et al., 2014). This physiological activity throughout the period when post oak has no leaves (less shade, no transpiration) may afford eastern redcedar the opportunity to persist and grow when competing with post oak even though its leaf-level rates of photosynthesis are lower during the growing season.

The Cross Timbers is located in the climate transition zone between forest and grassland. Climate is highly variable and the frequency and severity of episodic droughts are predicted to increase (Cook et al., 2015). Drought tolerance has been and will be necessary for long term survival of trees in this ecoregion. We did not find any differences in responses of the two drought-tolerant species to moderate drought. Eastern redcedar likely will continue to encroach into Cross Timbers forests, in the absence of fire, which will alter water budgets, carbon cycling, wildlife habitat, and have a broader social and economic impact (Joshi et al., 2019). However, severe drought may alter species competitive interactions, resulting in different outcomes and perhaps even cause differential mortality (Johnson et al., 2018).

## Data Availability Statement

The datasets for this article are not publicly available because some data are still in use for further publications. Requests to access the datasets should be directed to rodney.will@okstate.edu.

## Author Contributions

PT conceived of the study idea, developed study design, collected and processed the data, performed the analyses, and wrote the paper. CZ and RW conceived the study idea, developed the study design, helped the analysis and discussion of the results, and co-wrote the paper. AA and HA helped with the evaluation and review of the results and co-wrote the paper.

## Funding

This work was supported with funding from the National Science Foundation (NSF) under Grant No. OIA-1301789, Oklahoma Agricultural Experiment Station and McIntire-Stennis projects OKL0 2931 and OKL0 2929.

## Conflict of Interest

The authors declare that the research was conducted in the absence of any commercial or financial relationships that could be construed as a potential conflict of interest.
